# A rare case of long-term joint swelling caused by pigmented villonodular synovitis in a 3-year-old girl: a case report

**DOI:** 10.3389/fsurg.2023.1075171

**Published:** 2023-05-11

**Authors:** Yang Liu, Shaohua Liang, Wen Wang

**Affiliations:** ^1^ Department of Orthopedics, Guangzhou Red Cross Hospital of Jinan University, Guangzhou, China; ^2^ Guizhou Medical University, School of Clinical Medicine, Guiyang, China

**Keywords:** pigmented villonodular synovitis (PVNS), tenosynovial giant cell tumor (TGCT), arthroscopic synovectomy, knee, child

## Abstract

The clinical symptoms of pigmented villonodular synovitis (PVNS) are usually insidious and non-specific; therefore, delays in diagnosis and treatment are common. Here, we describe a case of a 3-year-old patient presenting with long-term joint swelling to highlight the significance of considering PVNS as a differential diagnosis in children to prevent misdiagnosis and ensure early treatment. After arthroscopic debridement, our patient had a favorable clinical outcome and was free of recurrence.

## Introduction

Pigmented villonodular synovitis (PVNS) is a rare, benign proliferative disease of the synovial joint, synovial bursa, and tendon sheath. The incidence rate of PVNS is approximately 1.8–8 cases per million people (1). Although it is usually observed in adults aged 20–50 years, PVNS also occurs in children in rare cases (2).

The symptoms of PVNS are usually insidious and non-specific. Swelling and pain are the major symptoms, and no specific finding is noted in the initial conventional radiography that is performed. Therefore, the diagnosis is often delayed or confused with hemophilic arthropathy, tuberculosis, juvenile idiopathic arthritis, and other rheumatological and malignant disorders.

## Case presentation

**Figure 1:**
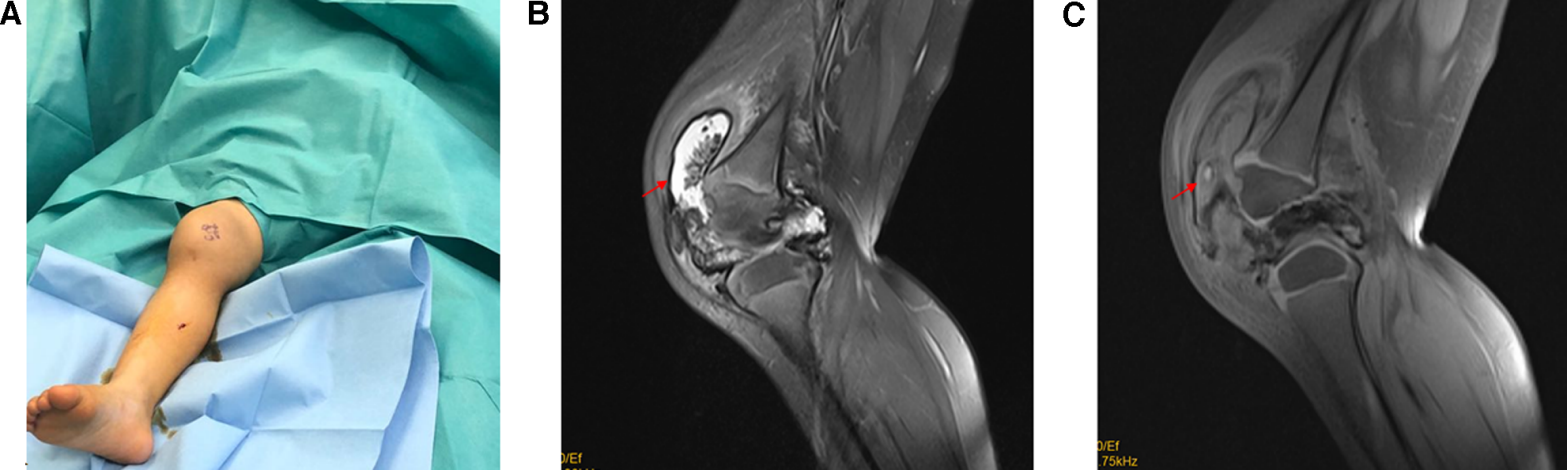
(**A**) Photo of right knee swelling. (**B**) Sagittal T2-weighted images illustrating a synovial lesion in the suprapatellar bursa. The arrow indicates scattered low-signal intensity areas in the villous hyperplastic synovium, representing hemosiderin deposits. (**C**) Sagittal T1-weighted fat-suppressed images illustrating bone erosion in the anterior aspect of the medial femoral condyle.

A 3-year-old girl presented with a history of swelling and pain in the right knee without a history of trauma, for 2 years (Figure 1A). The pain typically appeared after extensive walking or during stair climbing, while it disappeared at rest. A physical examination revealed tenderness near the insertion of the patellar tendon and proximal patellar tendon in the right knee. The patient reported a symptom of right knee swelling, and no palpable mass was noted. The range of motion in both knees was 0°–140°. The patient reported no familial history of rheumatological conditions or hemophilia. Based on symptoms and laboratory tests, bleeding disorder, rheumatoid arthritis, and infections were ruled out. A radiographic examination showed signs of swelling in the soft tissue around the knee joint. Magnetic resonance imaging (MRI) showed a large synovial mass posterior to the anterior patellar facet with femoral erosion (Figures 1B, C). The patient attempted to relieve the pain through traditional Chinese medicine and massage therapy prior to hospitalization, but the effects of these treatments were limited.

A modified multidirectional arthroscopic excision was planned for the patient. MRI showed a tumor-like mass, although the evidence of it being malignant was limited. Therefore, a preoperative biopsy was not performed to avoid overtreatment and clinical inconsequence.

**Figure 2:**
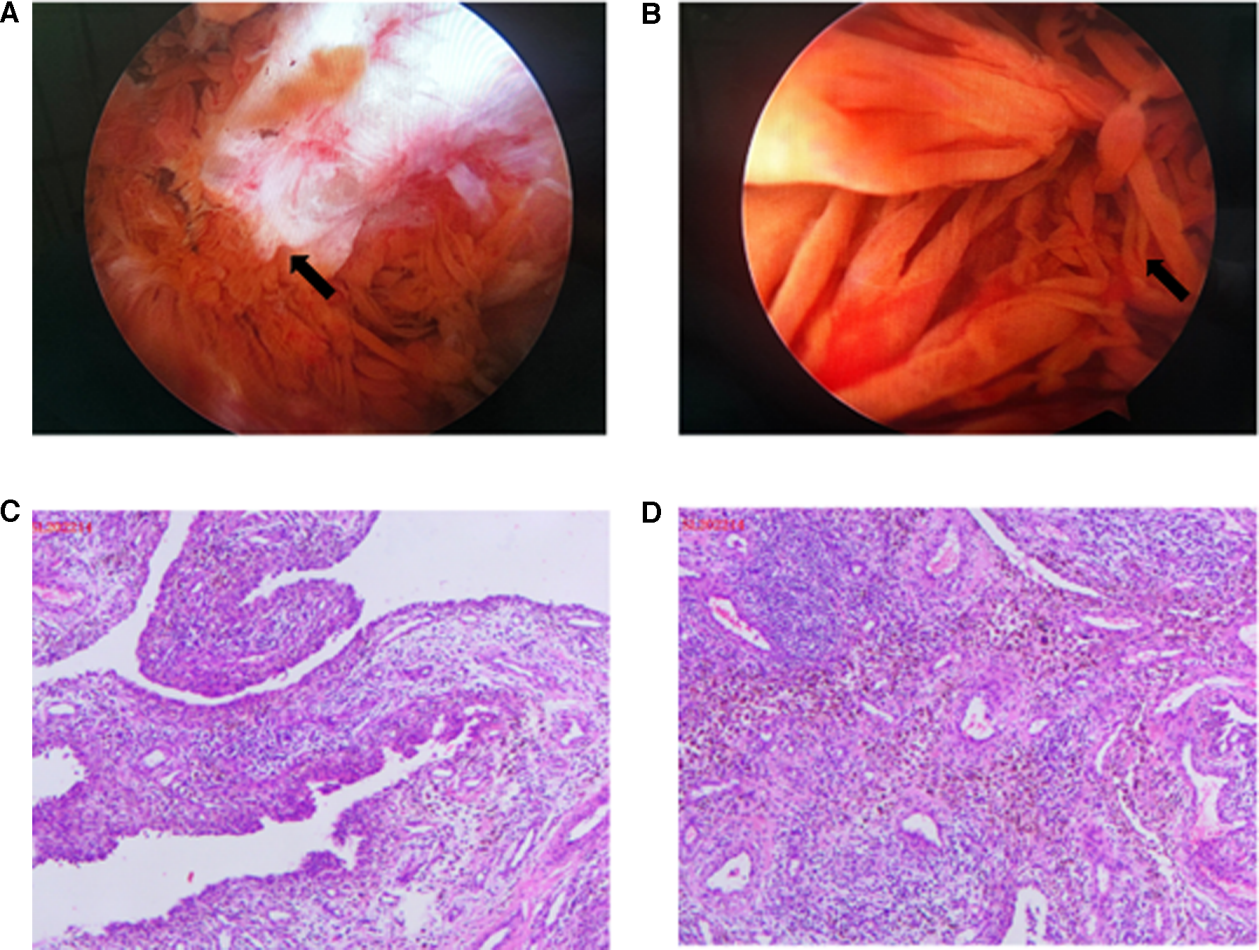
(**A**) Intraoperative arthroscopic pictures of bone erosion in the anterior aspect of the medial femoral condyle (arrow), which had been captured in MRI. (**B**) Intraoperative arthroscopic pictures demonstrating synovial proliferation (arrow) suggestive of pigmented villonodular synovitis. (**C, D**) Histology of the excised synovial tissue presenting characteristic PVNS features (immunocytochemistry CD68-positive).

A conventional anterolateral and anteromedial arthroscopic approach was adopted. After excising the synovium by using a motorized shaver, a well-defined yellow mass covered by the synovium was observed laterally adjacent to the distal patella (Figure 2A). Radiofrequency was used to loosen the nodule completely. The diseased synovium in the suprapatellar bursa, articular surface of the patella, and medial and lateral recesses was removed by performing radiofrequency coagulation. The diseased synovium in the posterior chamber and posterior aspect of the tibial plateau was removed using the posteromedial and posterolateral approaches (Figure 2B). The posterior chamber was comprehensively explored, and subsequently, synovial lesions in the posterior joint capsule could be removed completely. A histological examination of the nodule tissue exhibited synovial hyperplasia, foamy macrophages, spindle cells, multinucleated giant cells, and a considerable amount of hemosiderin deposits, which confirmed a diagnosis of PVNS (Figures 2C, D).

**Figure 3:**
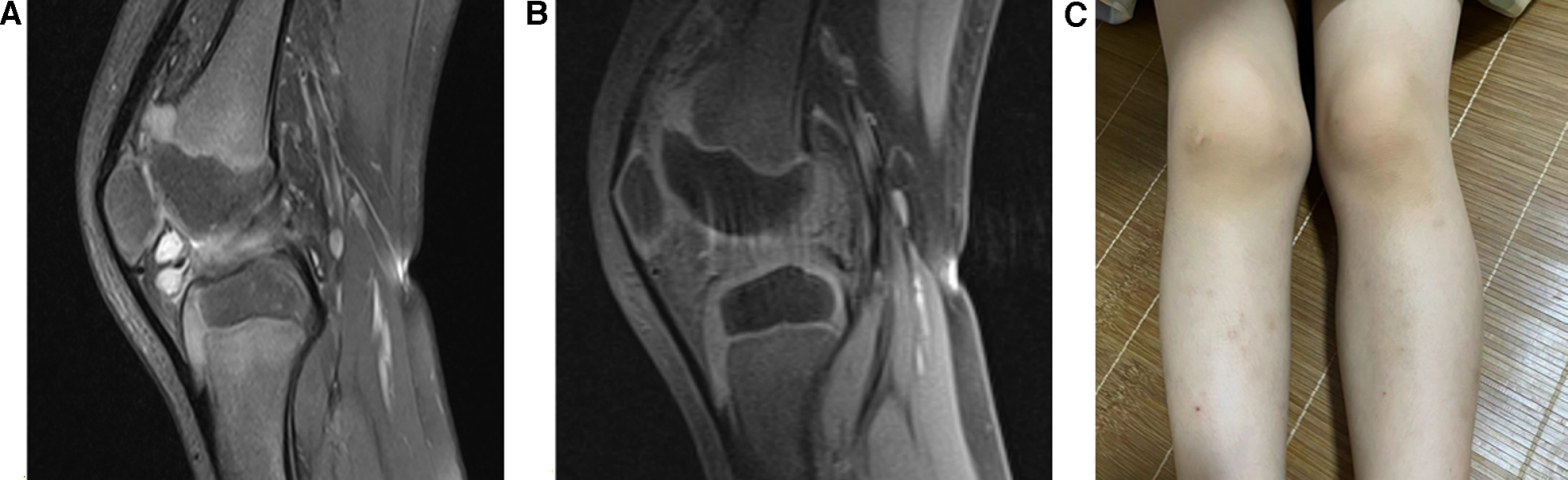
(**A, B**) MRI images at postoperative 12 months. Sagittal T2-weighted image and sagittal T1 fat compression image. Illustrating the disappearance of a cystic villous hyperplastic synovial lesion without recurrence, with a slight synovial thickening. (**C**) Photograph of a postoperative knee at 24 months.

The swollen knee was brought back to shape immediately after arthroscopic synovectomy. The child returned to the clinic 12 months after the surgery. MRI revealed no signs of recurrence (Figures 3A, B). At the second follow-up 24 months after the surgery, the child resumed her regular school life without reporting any knee pain (Figure 3C).

## Discussion

PVNS, which is characterized by its capacity to invade the subchondral bone and produce cysts and erosions, is rarely observed in children. It usually presents as swelling and pain in the affected joint (2). Mechanical complications such as meniscal injury and patellar dislocation are rare. While delayed diagnosis and surgery are correlated to poor functional outcome, early surgical excision and timely intervention could help improve the final outcome. The aim of surgery in the pediatric population is to remove abnormal tissue to relieve pain and reduce the risks of joint destruction and recurrence.

However, the established prognosis factors related to PVNS recurrence is limited. In a multicenter study (3), a large nodule size (>5 cm) and a prior recurrence were reported to be related to recurrence. Although open arthrotomy and complete synovectomy are associated with a reduced recurrence in PVNS patients, arthroscopic excision, which is preferred to open excision, also shows a high rate of efficacy for localized knee PVNS (4, 5). Furthermore, open arthrotomy and arthroscopic surgery showed no difference in local recurrence for both localized and diffuse PVNS in a meta-analysis of 1,019 participants (6). Compared with open excision, arthroscopic procedures are associated with a lower occurrence of postoperative complications and are therefore suggested for the initial treatment of localized PVNS (7). In addition, open excision may lead to an increased risk of osteoarthritic changes caused by the PVNS disease process, surgical invasion, and postoperative muscle weakness (8, 9).

The indication for arthroscopic excision should be considered comprehensively, including factors such as growth pattern and clinical behavior. There are two common types of PVNS with distinct characteristics, namely, rarely recurring localized PVNS and diffuse PVNS with a relatively high recurrence rate (10). Arthroscopic assessments of nodules located in the posterior compartment are complicated in nature. The uncommonly used portals may put the neurovascular bundle at risk, which might lead to an incomplete excision and would therefore require a further open synovectomy (11). Despite extensive information provided by arthroscopy techniques, ensuring a complete excision of the diseased synovium may prove to be a challenge to a less-experienced practitioner. Thus, open excision is still widely applied in treating the disease, and surgeons should make decisions in accordance with specific conditions. In the pediatric population, the minimally invasive nature of arthroscopy might be related to a more rapid recovery of joint function postoperatively and thus improve clinical outcomes.

Radiotherapy and isotopic synoviorthesis have been used in adults during relapse events or as adjuvant modalities (10). However, the use of radiotherapy in children is controversial due to the potential postirradiation sarcoma and damage to the epiphyseal growth plate (1, 12). Because PVNS exhibits characteristic cytogenetic abnormalities resulting in an overexpression of colony stimulating factor 1 (CSF1), systemic medication targeting the CSF1/CSF1R axis (imatinib, nilotinib, emactuzumab, and PLX3397) has been proposed in patients with diffuse, relapsed, or multifocal PVNS/TGCT (13–15). A clinical trial on pexidartinib conducted among adults paved the way for FDA clearance. However, such clinical trials specifically target those aged above 18 years. Evidence on the efficacy in children is lacking, warranting further investigation.

In summary, PVNS is a rare disease that mainly involves the knee, especially among children. Despite its rarity, PVNS should be considered as a differential diagnosis in a child presenting with monoarticular arthritis and no remarkable routine examination finding. To confirm the diagnosis, MRI is useful in terms of prompt diagnosis and determination of the severity of the lesion to ensure a better prognosis. Compared with the commonly used open arthrotomy, arthroscopic excision of the knee PVNS in children might lead to a more favorable outcome.

## Data Availability

The original contributions presented in the study are included in the article, and further inquiries can be directed to the corresponding author.

## References

[1] Stephan SR Shallop B Lackman R Kim TW Mulcahey MK. Pigmented villonodular synovitis: a comprehensive review and proposed treatment algorithm. JBJS Rev. (2016) 4(7):e3. 10.2106/JBJS.RVW.15.00086 27509331

[2] Karami M Soleimani M Shiari R. Pigmented villonodular synovitis in pediatric population: review of literature and a case report. Pediatr Rheumatol Online J. (2018) 16(1):6. 10.1186/s12969-018-0222-4 29343257 PMC5772710

[3] Mastboom MJL Staals EL Verspoor FGM Rueten-Budde AJ Stacchiotti S Palmerini E Surgical treatment of localized-type tenosynovial giant cell tumors of large joints: a study based on a multicenter-pooled database of 31 international sarcoma centers. J Bone Joint Surg Am. (2019) 101(14):1309–18. 10.2106/JBJS.18.01147 31318811

[4] Jain JK Vidyasagar JV Sagar R Patel H Chetan ML Bajaj A Arthroscopic synovectomy in pigmented villonodular synovitis of the knee: clinical series and outcome. Int Orthop. (2013) 37(12):2363-9. 10.1007/s00264-013-2003-5 PMC3843212 23860791

[5] Noailles T Brulefert K Briand S Longis PM Andrieu K Chalopin A Giant cell tumor of tendon sheath: open surgery or arthroscopic synovectomy? A systematic review of the literature. Orthop Traumatol Surg Res. (2017) 103(5):809–14. 10.1016/j.otsr.2017.03.016 28428036

[6] Aurégan JC Klouche S Bohu Y Lefèvre N Herman S Hardy P. Treatment of pigmented villonodular synovitis of the knee. Arthrosc J Arthrosc Relat Surg. (2014) 30(10):1327–41. 10.1016/j.arthro.2014.04.101 24999007

[7] Dines JS DeBerardino TM Wells JL Dodson CC Shindle M DiCarlo EF Long-term follow-up of surgically treated localized pigmented villonodular synovitis of the knee. Arthroscopy. (2007) 23(9):930–7. 10.1016/j.arthro.2007.03.012 17868831

[8] Nakahara H Matsuda S Harimaya K Sakamoto A Matsumoto Y Okazaki K Clinical results of open synovectomy for treatment of diffuse pigmented villonodular synovitis of the knee: case series and review of literature. Knee. (2012) 19(5):684–7. 10.1016/j.knee.2011.12.002 22264713

[9] van der Heijden L Mastboom MJ Dijkstra PD van de Sande MA. Functional outcome and quality of life after the surgical treatment for diffuse-type giant-cell tumour around the knee: a retrospective analysis of 30 patients. Bone Joint J. (2014) 96-B(8):1111–8. 10.1302/0301-620X.96B8.33608 25086129

[10] Fang Y Zhang Q. Recurrence of pigmented villonodular synovitis of the knee: a case report with review of literature on the risk factors causing recurrence. Medicine (Baltimore). (2020) 99(16):e19856. 10.1097/MD.0000000000019856 32312009 PMC7220429

[11] Georgiannos D Boutsiadis A Agathangelidis F Papastergiou S Karataglis D Bisbinas I. Arthroscopically-assisted mini open partial synovectomy for the treatment of localized pigmented villonodular synovitis of the knee. A retrospective comparative study with long-term follow up. Int Orthop. (2017) 41(5):925–30. 10.1007/s00264-016-3348-3 27866235

[12] Baroni E Russo BD Masquijo JJ Bassini O Miscione H. Pigmented villonodular synovitis of the knee in skeletally immature patients. J Child Orthop. (2010) 4(2):123–7. 10.1007/s11832-009-0236-z 21455469 PMC2839859

[13] Brahmi M Vinceneux A Cassier PA. Current systemic treatment options for tenosynovial giant cell tumor/pigmented villonodular synovitis: targeting the CSF1/CSF1R axis. Curr Treat Options Oncol. (2016) 17(2):10. 10.1007/s11864-015-0385-x 26820289

[14] Benner B Good L Quiroga D Schultz TE Kassem M Carson WE Pexidartinib, a novel small molecule CSF-1R inhibitor in use for tenosynovial giant cell tumor: a systematic review of pre-clinical and clinical development. Drug Des Devel Ther. (2020) 14:1693–704. 10.2147/DDDT.S253232 32440095 PMC7210448

[15] Nicholas G Bernthal NM Bukata SV Singh AS. Tenosynovial giant cell tumor: case report of a patient effectively treated with pexidartinib (PLX3397) and review of the literature. Clin Sarcoma Res. (2018) 8(1):1–5. 10.1186/s13569-018-0087-9 30002809 PMC6038319

